# Assessments of TP53 and CTNNB1 gene hotspot mutations in circulating tumour DNA of hepatitis B virus-induced hepatocellular carcinoma

**DOI:** 10.3389/fgene.2023.1235260

**Published:** 2023-08-01

**Authors:** Sonu Kumar, Neeti Nadda, Afnan Quadri, Rahul Kumar, Shashi Paul, Pranay Tanwar, Shivanand Gamanagatti, Nihar Ranjan Dash, Anoop Saraya, Shalimar, Baibaswata Nayak

**Affiliations:** ^1^ Department of Gastroenterology, All India Institute of Medical Sciences, New Delhi, India; ^2^ Laboratory Oncology Unit (BRA-IRCH), All India Institute of Medical Sciences, New Delhi, India; ^3^ Radiodiagnosis, All India Institute of Medical Sciences, New Delhi, India; ^4^ Gastrointestinal Surgery, All India Institute of Medical Sciences, New Delhi, India

**Keywords:** HBV, HCC, CtDNA, ddPCR, driver mutation, circulatory tumor DNA (ctDNA), chronic hepatitis B (CHB)

## Abstract

**Background:** Hepatitis B virus (HBV) infection is one of the major causes of chronic liver disease, which progresses from chronic hepatitis B (CHB) to fibrosis, cirrhosis, and hepatocellular carcinoma (HCC). Early detection and laboratory-based screening of hepatocellular carcinoma are still major challenges. This study was undertaken to determine whether the cancer hallmark gene signatures that are released into circulation as circulating tumour DNA (ctDNA) can be used as a liquid biopsy marker for screening, early detection, and prognosis of HCC.

**Methods:** A total of 130 subjects, including HBV-HCC (*n* = 80), HBV-cirrhotic and non-cirrhotic (*n* = 35), and healthy (*n* = 15) controls, were evaluated for TP53 and beta-catenin (CTNNB1) gene hotspot mutations in ctDNA by Sanger-based cycle sequencing and droplet digital PCR (ddPCR) assays. Mutation detection frequency, percentage mutant fractions, and their association with tumour stage, mortality, and smoking habits were determined.

**Results:** Sanger-based cycle sequencing was carried out for 32 HCC patients. Predict SNP Tools analysis indicated several pathogenic driver mutations in the ctDNA sequence, which include p.D228N, p.C229R, p.H233R, p.Y234D, p.S240T, p.G245S, and p.R249M for TP53 gene exon 7 and p.S33T for CTNNB1 gene exon 3. The TP53 c.746G>T (p.R249M) mutation was detected predominately (25% cases) by sequencing, but there was no dominant mutation at position c.747G>T (p.R249S) that was reported for HBV-HCC patients. A dual-probe ddPCR assay was developed to determine mutant and wild-type copy numbers of TP53 (p.R249M and p.R249S) and CTNNB1 (p.S45P) and their percentage mutant fraction in all 130 subjects. The TP53 R249M and CTNNB1 S45P mutations were detected in 31.25% and 26.25% of HCC patients, respectively, with a high mutant-to-wild-type fraction percentage (1.81% and 1.73%), which is significant as compared to cirrhotic and non-cirrhotic patients. Poor survival was observed in HCC patients with combined TP53 and CTNNB1 gene driver mutations. The TP53 R249M mutation was also significantly (*p* < 0.0001) associated with smoking habits (OR, 11.77; 95% CI, 3.219–36.20), but not the same for the TP53 R249S mutation.

**Conclusion:** Screening of ctDNA TP53 and CTNNB1 gene mutations by ddPCR may be helpful for early detection and identifying the risk of HCC progression.

## Graphical Abstract

**Figure:**
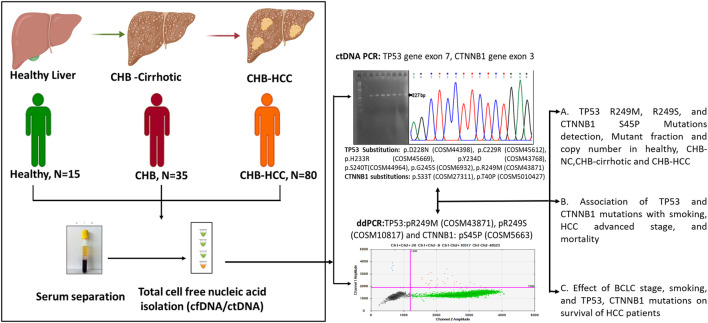
Created with BioRender.com.

## 1 Introduction

Hepatitis B virus (HBV) infection is implicated in acute and chronic hepatitis, cirrhosis, and hepatocellular carcinoma (HCC). As per the World Health Organization (WHO), 296 million people had chronic hepatitis B (CHB) infections in the year 2019, and about 1.5 million new infections are likely to occur each year (WHO, 2022). In the year 2019, 820,000 deaths were reported from HBV infections, which are mostly due to HBV-induced cirrhosis and HCC (WHO, 2022). As per WHO-IARC Globocan-2020 data, 4.7% new cases of liver cancer and 7.7% mortality due to liver cancer were reported globally (WHO, 2021b) whereas for India, 2.6% new cases and 4% mortality due to liver cancer were reported (WHO, 2021a). Major risk factor of HCC includes hepatitis B and C infections, cirrhosis, alcohol consumption, non-alcoholic fatty liver disease, aflatoxin exposure and tobacco smoking (Rumgay et al., 2022) which also corroborated for Indian patients as per Indian Council of Medical Research (ICMR) consensus document on HCC (Sirohi et al., 2020). Age-standardized mortality rate (ASMR) due to HCC in Indian population is reported to 6.8/100,000 population by ICMR consensus document (Sirohi et al., 2020). HBV and HCV infections are related to approximately 56% and 20% of primary liver cancer (Maucort-Boulch et al., 2018), respectively, whereas 18% and 17% of liver cancer burdens are related to tobacco smoking (Collaborators, 2020) and alcohol drinking globally, as per recent reports (Rumgay et al., 2021). Epidemiological evidence due to all causes shows that CHB accounts for 40% of HCC and 20%–30% of cirrhosis cases in India (Schmit et al., 2021).

The HBV belongs to the genus *Orthohepadnavirus* of family *Hepadnaviridae*. The viral genome consists of 3.2 Kb of partial double-stranded relaxed circular DNA (rcDNA), which encodes viral protein polymerase (P), core (C), surface (S), and X proteins (Seeger and Mason, 2015). During viral replication, three phases of HBV DNA occur, which include rcDNA, closed covalent circular DNA (cccDNA), and linear DNA. The viral genome may get integrated into the host genome or archived as cccDNA in its episomal form, increasing the risk of HCC (Lenhoff and Summers, 1994). The HBV-induced HCC may be attributed to persistent infection, viral genome integration and oncogenic potential of encoded viral proteins. Chronic inflammation and hepatocellular regeneration during CHB result in accumulations of genetic alterations leading to HCC (Nakamoto et al., 1998). There is an array of somatic driver mutations detected in HCC, irrespective of etiologies. In HBV-associated HCC, driver mutations were detected in several genes related to the cell-cycle pathway (TP53, ATM, RB1, CDKN2A), Wnt/β-catenin signalling pathway (CTNNB1, AXIN1, APC), telomere maintenance (TERT), epigenetic modification (ARID1A, 1B, and ARID2), oxidative stress (KEAP1, NFE2L2), PI3K/Akt/mTOR pathway, Ras/Raf/MAP pathway, and JAK/STAT pathways (Fujimoto et al., 2012; Guichard et al., 2012; Kan et al., 2013; Nault et al., 2014; Kawai-Kitahata et al., 2016).

Somatic driver mutations can be detected in circulating tumor DNA (ctDNA) samples from HCC patients (Huang et al., 2016). The ctDNAs belong to a small fraction of cell-free DNA (cfDNA), which carries tumour genetic information, including driver mutations and aberrant methylation (Labgaa and Villanueva, 2015). The cfDNAs, including ctDNAs, are released into the circulation through the processes of apoptosis, necrosis, and active secretion by tumor cells (Thierry et al., 2016). Recently, we have evaluated cfDNA concentration and integrity index in HCC and its possibilities as a liquid biopsy marker for early detection of HCC (Kumar et al., 2022). Liquid biopsy marker detection and analysis, including ctDNA, will be helpful for the detection of cancer at an early stage and also for the assessment of response to therapy, tumour recurrence, and drug resistance (Murtaza et al., 2013; Luke et al., 2014; Montagut et al., 2015; Heitzer et al., 2015; Reinert et al., 2016; Nadda et al., 2020). Targeted driver gene mutations or hotspot mutations in ctDNA are also studied by sanger sequencing of amplicons, nested PCR-RFLP, and highly sensitive droplet digital PCR (ddPCR) techniques to detect the frequency of ctDNA mutations in the background of abundant cfDNA (Marchio et al., 2018). In HBV-induced HCC, candidate genes of choice are TP53, CTNNB1, and TERT due to the prevalence of high-frequency driver mutations (Li and Mao, 2013; Wang et al., 2013; Totoki et al., 2014; Villanueva and Llovet, 2014; Schulze et al., 2015; Zucman-Rossi et al., 2015; Hirotsu et al., 2016).

In this study, we aim to detect the driver mutations in the ctDNA during different stages of CHB leading to HCC and whether these ctDNA driver mutations can be used as liquid biopsy marker for early detection of HCC. There is an acute need of biomarker which can be used for routine surveillance of HCC in CHB patients those are under clinical follow up and/or while testing HBV DNA load. Different hotspot or driver mutations may be detected in ctDNA with increased frequency in HCC patients or in CHB patients progressing cirrhosis and HCC. The ddPCR is highly sensitive for the detection of low-frequency ctDNA driver mutations in the background of a high load of cfDNA (Huerta et al., 2021). Due to the ease of this technique, it can be used for HCC surveillance or to study changes during HCC progression to advanced stages of HCC and can be tested in serial samples (Marchio et al., 2018).

## 2 Materials and methods

### 2.1 Study population and design

A total of 130 subjects were enrolled in this prospective observational study. Of these, 115 patients were related to HBV etiology, including HCC (*n* = 80), cirrhotic (*n* = 20), and non-cirrhotic (*n* = 15) CHB patients. These patients were recruited between February 2019 and December 2021 from the liver clinic of the Department of Gastroenterology at the All India Institute of Medical Sciences (AIIMS), New Delhi, India. Chronic HBV infection was established by the presence of HBsAg for at least 6 months (HBsAg, anti-HBc positive, and anti-HBs negative). Cirrhosis was diagnosed based on ultrasonography, elastography, and a liver biopsy, if available. HCC was diagnosed as per the European Association for the Study of the Liver (EASL) criteria (European Association for the Study of the LiverEuropean Association for the Study of the Liver, 2018) and staged as per BCLC classification (Bruix et al., 2011). Healthy volunteers (*n* = 15) included in this study as controls were serologically negative for anti-HCV, HBsAg, and anti-HBc. The study protocol was approved by the institute’s ethical committee as per IECPG-38/23.01.2019 and RT-13/28.02.2019. All participants were more than 18 years old and had given written consent. Exclusion criteria for patients include comorbidity with HIV and HCV, pregnancy, renal failure, and sepsis. The demographic profile and clinical and biochemical parameters for all patients were recorded.

### 2.2 Blood sample collection for genomic DNA and ctDNA isolation

Peripheral blood samples were collected in an EDTA vial and a plain vial (Levram Lifesciences, India) with a gel-clot activator for quick serum separation, taking precautions not to damage genomic DNA. The genomic DNA from whole blood was isolated using the QIAamp mini genomic DNA isolation kit (Qiagen), and ctDNA was isolated from serum samples using the QIAamp DSP mini nucleic acid isolation kit (Qiagen) in the QIAsymphony automated nucleic acid extraction system (Qiagen). Briefly, 400 μL of serum was used as the starting material, and the elution volume was 40 μL. The concentration of ctDNA was measured using a MultiSkanGo spectrophotometer (Thermofischer), and the ctDNA purity was analysed using the A260/A280 ratio.

### 2.3 PCR amplification of TP53 and CTNNB1 gene

Earlier studies in HCC patients have documented hotspot mutations in the exon 7 of the TP53 gene (DNA binding domain) (Tanaka et al., 1993; Kirk et al., 2000) and exon 3 of the CTNNB1 (β-catenin) gene (Lenhoff and Summers, 1994; Hsu et al., 2000). International Agency for Research on Cancer (IARC) recommended primers were used for amplification of TP53 exon 7 (P333, 510F-5′-CTTGCCACAGGTCTCCCCAA-3′, P313, 746R- 5′-AGGGGTCAGCGGCA AGCAGA-3′) and CTNNB1 exon 3 (444F-5′-GCTGATTTGATGGAGTTGGA-3′, 670R- 5′-GCT​ACT​TGT​TCT​TGA​GTG​AA-3′). PCR amplification was carried out in a 25 µL final reaction volume using a thermocycler (SureCycler 8800, Agilent Technologies). Each reaction mixture contained 3 µL of ctDNA template, 1.25 µL forward and reverse primers each (10 µM), 2.5 µL of 10X Dream Taq Buffer (Thermo-Scientific), 0.50 µL of 10 mM dNTP mix (Thermo-Scientific), 0.25 µL of Dream Taq polymerase (5 U/µL) (Thermo-Scientific), and 16.25 µL nuclease-free water (Qiagen). Thermocyler conditions were denaturation at 95°C for 5 min, followed by 50 cycles of denaturation at 94°C for 30 s, annealing (60°C, TP53, and 58°C, CTNNB1) for the 30 s, extension at 72°C for 30 s, and final extension at 72°C for 10 min. PCR products were kept at 4°C for further storage. Amplification of TP53 (237 bp) and β-catenin (227 bp) was confirmed by 2% agarose gel electrophoresis, and the desired bands were excised for gel purification.

### 2.4 Sequencing of TP53 and β-catenin gene

Fluorescence-based cycle sequencing of TP53 and CTNNB1 amplicons was carried out in a 10 μL volume using 2 μL of gel purified product, 1 μL sequencing primer, and 7 μL big dye terminator sequencing reaction mix. Thermal cycling was carried out at 95°C for 2 min for a hot start and 25 cycles of each denaturation at 95°C for 10 s, annealing at 55°C for 5 s, extension at 60°C for 4 min. The cycle sequencing products were subjected to capillary electrophoresis in ABI 3730XL DNA analyzer at the Eurofins-Scientific sequencing facility, Bangalore, India. The sequencing data files were processed using the Sequencing Analysis V5.3 software, and Seq Scanner was used to generate PDF and Fasta files from abi files. Nucleotide and deduced amino acid sequence alignment with reference and determination of mutation were carried out using DNASTAR software.

### 2.5 Functional annotation of missense variants, pathogenicity prediction, and impact on protein stability

The functional impact of non-synonymous single nucleotide polymorphisms (nsSNPs) or mutations on the tertiary structure of proteins was predicted using the amino acid sequence in FASTA format through online software. Predict SNP, MAPP, PhD-SNP, PolyPhen-1, PolyPhen-2, SIFT, and SNAP. Additionally, Provean, FATHMM, Panther-PSEP, SNPs & GO, and MutPred 2 tools were used to revalidate disease-related missense variants based on their pathogenicity. An alteration in protein stability due to nsSNPs was predicted by using the MUpro and I-Mutant 2.0 tools. The protein sequence was used as a predictor of the direction of protein stability or change in Gibb’s free energy (∆∆G = ∆G^WT^–∆G^M^). The positive and negative values of ∆∆G indicate increased protein stability as well as destabilization, respectively. The conservation of the residues at the SNP locations was predicted using the Consurf tool. Highly conserved residues were represented by higher scores, whereas variable residues were represented by lower scores closer to 1.

### 2.6 Frequency and detection of ctDNA driver mutations by droplet digital PCR (ddPCR) assay

The ddPCR Mutation Detection Assays for TP53 gene mutations in Exon 7 (p.R249M, c.746 G˃T, COSM43871 and p. R249S c.747G>T, COSM10817) and the CTNNB1 gene mutation in Exon 3 (p.S45P, c.133T>C, COSM5663) were designed for proprietary Droplet Digital™ PCR (ddPCR). All these three assays have been wet-lab validated by Bio-Rad under assay IDs: dHsaMDS2514856, dHsaMDV2010087 and dHsaMDS835681322. Each assay contains two probe conjugated with FAM targeting the mutant allele and HEX targeting the wild-type allele. The QX200 droplet digital PCR system was used for mutant detection according to the manufacturer’s instructions. The ddPCR reactions were carried out using 10 µL of 2X ddPCR (no UTP) Super-Mix (Bio-Rad), 9 μL ctDNA as a template, 1 µL of 20X dual-probe assay mix (Bio-Rad), having both wild type probe/primer mix and mutant probe/primer mix, and 3 deionized distilled water. Each 23 μL reaction volume was carefully loaded into the well of the droplet generator cartridge (Bio-Rad), and 70 μL droplet generation oil (Bio-Rad) was subsequently loaded to generate droplets. The cartridge was covered with Droplet Generator Gasket (Bio-Rad) and transferred into the QX200 Droplet Generator (Bio-Rad) to generate droplets from each sample. Then 40 μL droplets from each sample were transferred into a 96-well PCR plate for amplification. PCR was performed using the program: 95°C for 10 min, followed by 40 cycles of 94°C for the 30 s and 60°C (TP53: c.746 G˃T/TP53: c.747 G˃T/CTNNB1:133T>C) for 60 s, followed by 98°C for 10 min, and holding at 4°C for 30 min (PCR conditions were recommended in the assay data sheet). The rate of temperature rise was set at 2.5°C/s. After the PCR was done, the sealed 96-well plate was transferred to the QX200 Droplet Reader (Bio-Rad). The sealed plate with PCR product was read with the QX200 Droplet Reader (Bio-Rad) using QuantaSoft (Bio-Rad) software. All the further analysis to determine wild-type and mutant droplets was performed using QuantaSoft (Bio-Rad) software. The set threshold is depicted in Figure 2 that is 1,900 for TP53 p.R249M, c.746 G˃T, COSM43871 and CTNNB1gene p. S45P, c.133T>C. COSM5663 and 12,000 for TP53 p.R249S c.747G>T, COSM10817.

### 2.7 Statistical analysis

Statistical analysis was performed using GraphPad Prism 9.4.1 software. All the parametric data were expressed as mean ± SD and non-parametric data was expressed as median with interquartile range (IQR). The significance of the data (*p* < 0.05) was analyzed using one-way ANOVA, Kruskal-Wallis test. Dunn’s multiple comparisons were done within groups, and the significance among the groups is depicted within the graphs. The association of TP53 and CTNNB1 mutations with smoking habits, advanced tumor stage, and mortality was analyzed by the chi-square test. The statistical significance of survival plots related to mutation, smoking, and early or advanced HCC was obtained using the log-rank test and the hazard ratio by the Mantel-Haenszel test.

## 3 Results

### 3.1 Demographic, clinical, and biochemical profiles of the study population

The demographic profile and the clinical and biochemical parameters of the healthy control, CHB-HCC, CHB-cirrhotic, and CHB-noncirrhotic (CHB-NC) patients are mentioned in Table 1. The mean age of CHB-HCC patients (52.95 ± 12.74 years) and CHB-cirrhotic (47.9 ± 11.5 years) patients was higher than that of CHB-NC patients (38.45 ± 10.87 years) and healthy controls (38.8 ± 6.28 years). There was a male predominance in both CHB (83%) and HCC (86.25%) cases. The smoking habit was observed in 34% of CHB and 20% of HCC patients. Tumor characteristics including tumor size, child’s score, BCLC staging, PST score, and AFP production, were mentioned in Table 1.

**TABLE 1:** Comparison of demographic, biochemical, and clinical profile among healthy, CHB and HBV-HCC patients.

Sr. No	Variable	Healthy (*n* = 15)	CHB-noncirrhotic (*n* = 15)	CHB-cirrhotic (n = 20)	HCC (*n* = 80)	*p*-value
1	Age (years)	38.8 ± 6.28	38.45 ± 10.87	47.9 ± 11.5	52.95 ± 12.74	**0.0001**
2	Sex (Male, n %)	11 (73.3%)	12 (83%)	17 (85%)	69 (86.25%)	0.10
3	Smoking (Smoker %)		5 (33.3%)	7 (35%)	16 (20%)	0.29
4	HBV Etiologies (%)	-	15 (100%)	20 (100%)	80 (100%)	0.99
5	HBV DNA at baseline	-	12 (80%)	13 (65%)	25 (31.25%)	**0.002**
6	HBsAg positive	-	14 (93.3%)	18 (90%)	75 (93.75%)	0.21
7	HBeAg Positive	-	9 (60%)	4 (20%)	23 (28.75%)	0.31
8	Child’s Score					NA
	A	-	-	-	47 (58.75%)	
	B	-	-	-	27 (33.75%)	
	C	-	-	-	6 (7.5%)	
9	BCLC Stage			-		NA
	**0**	-	-	-	1 (1.25%)	
	A	-	-	-	17 (21.25%)	
	B	-	-	-	24 (30%)	
	C	-	-	-	29 (36.25%)	
	D				9 (11.25%)	
10	PST Score					NA
	0	-	-	-	38 (47.5%)	
	1	-	-	-	34 (42.5%)	
	2	-	-	-	04 (5%)	
	3	-	-	-	04 (5%)	
11	Base line Tumor Size	-	-	-	6.14 ± 3.92	NA
	**>3 cm**				20 (25%)	
	**<3 cm**				60 (75%)	
	Single Tumor				41 (51.25%)	
	Multiple Tumor				39 (48.75%)	
12	Type II Diabetes Mellitus (*n*, %)	-	1 (6.6%)	2 (8%)	12 (15%)	0.09
13	Cirrhosis	-	0 (0%)	20 (100%)	71 (89%)	0.16
14	AFP (ng/mL)		2.6 ± 0.68	14.4 ± 21.5	12,262.1 ± 46,705.4	**0.001**
	<20 (ng/mL)	-	15 (100%)	17 (85%)	24 (30%)	
	>20 (ng/mL)	-	-	3 (15%)	56 (70%)	
15	Hb (gram/dL)	-	13.8 ± 2.03	12.98 ± 1.09	11.7 ± 2.07	0.17
16	TLC (per mm^3^)	-	8013 ± 3,760	7,615 ± 2065	7,162.5 ± 3,016.6	0.47
17	Platelets (n*10^3^)	-	172.7 ± 83.1	161.8 ± 67.6	143.1 ± 69.6	0.08
18	Serum Albumin (g/dL)	-	4.06 ± 1.09	3.9 ± 1.14	3.56 ± 0.73	0.057
19	Bilirubin (mg/dL)	-	1.10 ± 0.89	1.9 ± 4.1	1.74 ± 3.82	0.83
20	AST (IU/mL)	-	133.6 ± 231	52.7 ± 22.22	86.1 ± 62.72	0.06
21	ALT (IU/mL)	-	81.27 ± 113.3	52.35 ± 23.14	63.5 ± 44.74	0.34
22	SAP (IU/mL)	-	237 ± 143	189.7 ± 92.17	336.6 ± 372.12	0.15
23	Total Protein (g/dL)	-	7.2 ± 0.95	7.02 ± 0.57	7.3 ± 0.81	0.78

Bold *p*-value indicates statical significance (*p* < 0.05).

**Abbreviations:** AST, aspartate aminotransferase; ALT, alanine aminotransferase; SAP, serum alkaline phosphatase; Hb, Haemoglobin; TLC, total leucocyte count; PLT, platelet count; AFP, Alpha-fetoprotein; HBV, Hepatitis B virus.

### 3.2 Cycle sequencing of TP53 and CTNNB1 amplicons and bioinformatic based pathogenicity prediction of the dominant driver mutation of HCC

**FIGURE 1:**
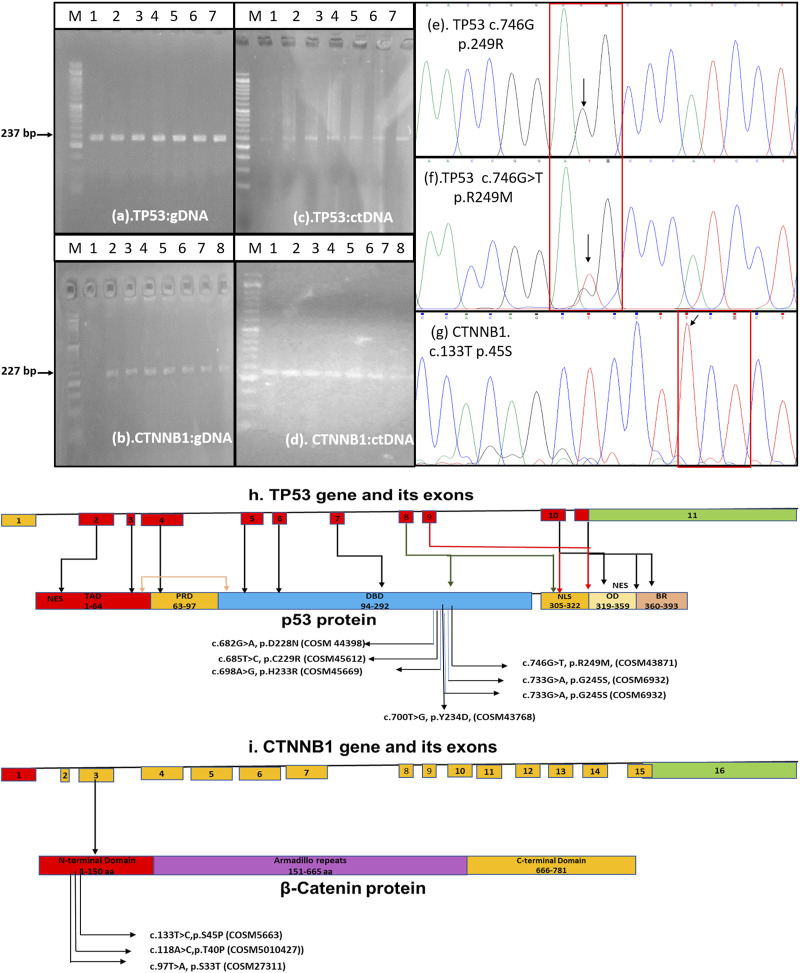
Amplification of TP53 gene exon 7 and CTNNB1 gene exon 3 from genomic DNA **(A,B)** and ctDNA **(C,D)** for fluorescence-based cycle sequencing Color chromatograph depicting the TP53-c.746G, p. R249-WT **(E)**, TP53-c.746G>T, p. R249M-MT **(F)**, and CTNNB1-c.133T **(G)** regions. **(H)** Schematic of TP53 gene with substitutions reported in our study and its cosmic data base annotation. The TP53 coding region consists of 9 (red boxes) out of 11 exons. The annotated cosmic ID of substitutions in the exon 7 corresponding to DNA binding domain (DBD) has been shown in schematics. Other domains of TP53 includes Transactivating domain (TAD), Proline Rich Domain (PRD), Nuclear Localization Signal (NLS), Oligomerization Domain (OD), and Basic Repression (BR) domain. **(I)**The schematics of CTNNB1 gene which contains 16 exons which encodes 781 aa long beta -catenin protein. The substitutions in the exon 3, its annotation and COSMIC ID is mentioned in the schematic.

PCR amplification with the desired band size was carried out for both the TP53 gene flanking exon 7 (237 bp) and the CTNNB1 gene flanking exon 3 (227 bp) in 32 out of 80 HCC patients (Figure 1). Fluorescence-based cycle sequencing of these amplicons was carried out, and the nucleotide sequence data along with the colour chromatogram (Figure 1) were obtained. Nucleotide and deduced amino acid sequence alignment with the reference sequence was carried out for the determination of mutation. The TP53 nucleotide sequences of 32 patients were submitted to Genbank with accession numbers ranging from OR232241 to OR232272, whereas the CTNNB1 sequences of patients were deposited at Genbank with accession numbers ranging from OR232273 to OR232304.

Non-synonymous mutations were detected in the TP53 gene exon 7 at positions c.678G>A (p.D228N), c.682T>C (p.C229R), c.695A>G (p.H233R), c.698T>G (p.Y234D), c.718G>C (p.S240T), c.732G>A (p.G245S), and c.746G>T (p.R249M). No dominant mutation at TP53 position c.747G>T (p.R249S) was observed. The frequency of mutations ranged from 3.1% to 25% among HCC patients. The mutation TP53 c.746G>T (p.R249M) was detected more frequently (25% cases) in HCC patients by sequencing. Most of these mutations were found pathogenic through different prediction software, as mentioned in Table 2. Using SNP tools like MAPP, PhD-SNP, PolyPhen-1, PolyPhen-2, SIFT, SNAP, Provean, FATHMM, Panther-PSEP, SNPs & GO, and MutPred2, the nature of SNPs as deleterious, neutral, benign, disease-causing, and cancer-causing was determined with predicted percentages that ranged from 51% (TP53, pD228N) to 87% (TP53, pR249M). MUpro and I-mutant analyses of these nsSNPs indicated lower structural stability due to these mutations. Consurf tools identified these mutations in structurally and functionally conserved residues of the TP53 native protein with a high score for all except one nsSNP for the D229A position with a score of 1 (Table 2).

**TABLE 2:** Sanger sequencing of TP53 gene exon 7 for non-synonymous single nucleotide polymorphisms (nsSNP) mutation frequency and pathogenicity prediction using different bioinformatics tools.

Tool	c.682G>A p.D228N	c.685T>C p.C229R	c.698A>Gp.H233R	c.700T>Gp.Y234D	c.719G>C p.S240T	c.733G>A p.G245S	c.746G>T p.R249M
Cosmic ID	COSM 44398	COSM45612	COSM45669	COSM43768	COSM44964	COSM6932	COSM43871
Frequency	3.125% (1/32)	3.125% (1/32)	3.125% (1/32)	3.125% (1/32)	3.125% (1/32)	3.125% (1/32)	25% (8/32)
MutPred2	0.198	0.411	0.482	0.817	0.243	0.546	0.552
PredictSNP	D 51%	D 65%	D 87%	D 76%	D 52%	D 72%	D 87%
MAPP	D 75%	D 86%	D 63%	D 84%	N 74%	D 57%	D 81%
PhD-SNP	D 59%	D 77%	D 68%	D 88%	D 73%	D 88%	D 88%
PolyPhen-1	N 67%	D 59%	D 59%	D 74%	N 67%	N 67%	D 74%
PolyPhen-2	N 70%	D 40%	D 63%	N 61%	D 47%	D 81%	D 81%
SIFT	D 79%	N 74%	D 53%	D 79%	D 45%	D 79%	D 79%
SNAP	D 61%	D 62%	D 72%	D 81%	D 62%	D 62%	D 85%
PROVEAN	N	D	N	D	D	D	D
FATHMM	Cancer	Cancer	Cancer	Cancer	Cancer	Cancer	Cancer
PANTHER	Benign	Cancer	Benign	Benign	Benign	Benign	Benign
SNPs & GO	Disease	Disease	Disease	Disease	Disease	Disease	Disease
I-Mutant DDG, Stability	−0.42↓	−0.78↓	−0.8↓	−0.95↓	−0.39↓	−0.05↓	−0.85↓
Mu-Pro DDG, Stability	−0.87↓	−1.04↓	−0.84↓	−0.67↓	−0.48↓	−0.8↓	−0.95↓
Consurf Score	7	1	9	8	9	9	9

Non-synonymous mutations detected by sequencing of the CTNNB1 gene exon 3 are mentioned in Table 3 at positions CTNNB1 c.97T>A (p.S33T), c.118A>C (p.T40P), c.155C>A (p.P52H), c.161A>C (p.E54A), c.163G>C (p.E55Q), and c.171G>A (p.V57M). The frequencies of these mutations were very low (Table 3). The earlier reported dominant mutation, c.133T>C (p.S45P), was not detected by sequencing in our HCC patients. The pathogenic prediction software found only CTNNB1, c.97T>A p.S33T SNP, as deleterious, and other mutants were predicted to be neutral (Table 3).

**TABLE 3:** Sanger sequencing of CTNNB1 gene exon 3 for non-synonymous single nucleotide polymorphisms (nsSNP) mutation frequency and pathogenicity prediction using different bioinformatics tools.

Tool	c.97T>A p.S33T	c.118A>C p.T40P	c.155C>A p.P52H	c.161A>C p.E54A	c.163G>C p.E55Q	c.171G>A p.V57M
Cosmic ID	COSM27311	COSM5010427	-	**-**	**-**	**-**
Frequency	3.125% (1/32)	3.125% (1/32)	3.125% (1/32)	3.125% (1/32)	3.125% (1/32)	3.125% (1/32)
MutPred2	0.836	0.876	0.744	0.665	0.427	0.466
PredictSNP	D 61%	N 83%	N 83%	N 83%	N 83%	N 83%
MAPP	N 75%	N 64%	N 73%	No score	No score	No score
PhD-SNP	D 68%	N 72%	N 89%	N 83%	N 78%	N 89%
PolyPhen-1	D 74%	N 67%	N 67%	N 67%	N 67%	N 67%
PolyPhen-2	D 81%	N 68%	N 87%	N 87%	N 72%	N 79%
SIFT	D 79%	N 68%	N 78%	N 70%	N 66%	N 79%
SNAP	N 50%	N 77%	N 83%	N 67%	N 77%	N 83%
PROVEAN	Neutral	Neutral	Neutral	Neutral	Neutral	Neutral
FATHMM	Cancer	Cancer	Cancer	Cancer	Cancer	Cancer
PANTHER	No Score	No Score	No Score	No Score	No Score	No Score
SNPs & GO	Disease	Disease	Disease	Disease	Disease	Disease
I-Mutant DDG, Stability	0.29↑	−1.48↓	−1.16↓	−0.96↓	−1.05↓	−0.61↓
Mu-Pro DDG, Stability	−1.15↓	−1.18↓	−0.8↓	−0.9↓	−1.26↓	−1.18↓
Consurf Score	9	1	1	1	1	1

In the case of CTNNB1, only the S33T SNP displayed a deleterious nature by predictSNP with a prediction score of 61%. Additionally, FATHMM and SNPs & GO indicated that these mutants may be cancer-causing polymorphisms. I-mutant and MUpro analyses indicated that mutations at these positions may lower the structural stability of proteins. Consurf tools observed higher scores only for nsSNP (S33T), which indicates that it is more conserved than others (Table 3).

### 3.3 Detection of TP53 R249M, TP53 R249S, and CTNNB1 S45P mutations in healthy, CHB-NC,-cirrhotic and CHB-HCC patients by droplet digital PCR assay

**FIGURE 2:**
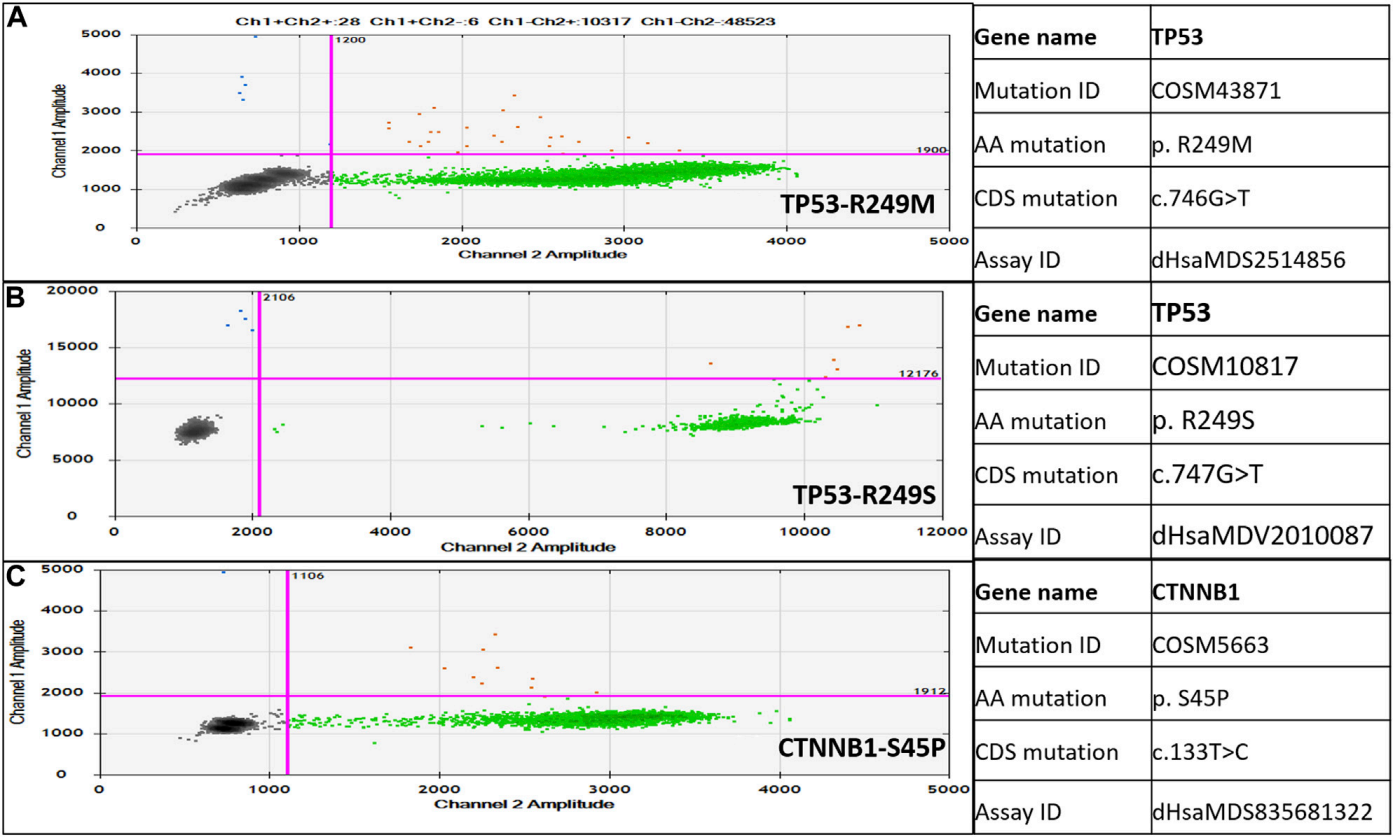
Droplet digital PCR (ddPCR)-based dual-probe assay for TP53-c.746G>T, p.R249M **(A)**, TP53-c.747G>T, p.R249S **(B)**, and CTNNB1-c.133T>C, p.S45P **(C)**. Mutation ID and assay ID are mentioned on the right side of the dot plot. Blue dots are droplets with mutant type alleles only; red droplets have mutant as well as wild type alleles; and green dots are droplets with wild type alleles only.

Detection of TP53 gene c.746G>T (p.R249M), c.747G>T (p.R249S), and CTNNB1 gene c.133T>C (p.S45P) mutations in healthy, CHB, and HCC patients was carried out by a dual-probe based ddPCR assay, which can detect both wild-type and mutant types (Figure 2). The ddPCR assay is highly sensitive and can detect mutant-type ctDNA in the background of wild-type cfDNA. TP53 and CTNNB1 mutations were detected in a higher percentage of HCC patients, followed by CHB-cirrhotic than CHB-NC, whereas no mutation was detected in healthy controls (Table 4). TP53, p.R249M mutation, was detected in a higher percentage (31.25%) of HCC patients compared to TP53, p.R249S mutation (13.75%). At least one driver mutation was detected in 47 out of 80 HCC patients (58.75%) by a dual-probe ddPCR assay (Table 4). The percentage of mutant-type was significantly higher in CHB-HCC and CHB-Cirrhotic than in CHB-NC and healthy controls (Table 4). In HCC patients, the TP53 mutant type was detected (42.5%) more than the CTNNB1 mutant type (26.25%).

**TABLE 4:** Mutation frequency percentage, wild type and mutant copy number, and mutant fraction percentage among healthy, CHB-noncirrhotic, CHB-cirrhotic and CHB-HCC patients.

Detection frequency	Healthy *n* = 15	CHB-NC *n* = 15	CHB-cirrhotic *n* = 20	CHB-HCC *n* = 80	*p*-Value
TP53 R249M, %	0 (0/15)	6.66 (1/15)	20 (4/20)	31.25 (25/80)	***p* = 0.01**
TP53 R249S, %	0 (0/15)	6.66 (1/15)	25 (5/20)	13.75 (11/80)	*p* = 0.14
TP53 R 249 M or S, %	0 (0/15)	13.33 (2/15)	30 (6/20)	42.5 (34/80)	***p* = 0.003**
CTNNB1 S45P, %	0 (0/15)	6.66 (1/15)	15 (3/20)	26.25 (21/80)	***p* = 0.01**
R 249 M/S + S45P,%	0 (0/15)	20 (3/15)	40 (8/20)	58.75 (47/80)	***p* < 0.0001**

Wild type (copies/mL)	Healthy *n* = 15	CHB-NC *n* = 15	CHB-Cirrhotic *n* = 20	CHB-HCC *n* = 80	
TP53-c.746G (p.249R)-WT	1725822 (821,674–9410433)	1954049 (875,676–3472375)	4071811 (2505278–8015181)	2332971 (1161798–4613364)	*p* = 0.060
TP53c.747G (p.249R)-WT	2004397 (400,551–3,2581818)	6671760 (3466884–18083034)	2915878 (1551136–5775551)	2165271 (1085008–6908540)	*p* = 0.064
CTNNB1-c.133T (p.45S)-WT	1729453 (628,821–2290454)	1196776 (768,879–1550142)	1991020 (927,157–3825264)	1812840 (884,273–5737849)	*p* = 0.102

Mutant (copies/mL)	Healthy *n* = 15	CHB-NC *n* = 15	CHB-Cirrhotic *n* = 20	CHB-HCC *n* = 47	
TP53-c.746G>T (p.R249M)-MT	0	3,250 ± 12,585	16,381 ± 37,169	41,467 ± 104,170	***p* = 0.0003**
TP53-c.747G>T (p.R249S)-MT	0	1188 ± 4,600	10,788 ± 20,619	23,654 ± 65,793	*p* = 0.09
CTNNB1-c.133T>C (p.S45P)-MT	0	336 ± 1301	3,173 ± 8153	29,063 ± 73,806	***p* = 0.0004**

Mutant Fraction (MT/WT), %	Healthy *n* = 15	CHB-NC *n* = 15	CHB-Cirrhotic *n* = 20	CHB-HCC *n* = 47	
TP53 R249M	0	0.1120 ± 0.4339	0.3385 ± 0.7468	1.814 ± 6.750	***p* = 0.0238**
TP53 R249S	0	0.03426 ± 0.1327	0.1950 ± 0.5267	0.9546 ± 3.104	*p* = 0.1005
CTNNB1 S45P	0	0.03426 ± 0.1327	0.1950 ± 0.5267	1.731 ± 7.793	***p* = 0.0022**

Bold values indicate significant *p* values.

### 3.4 Mutant fraction and copy number of wild type and mutant type in healthy, CHB, cirrhotic, and HCC patients

**FIGURE 3:**
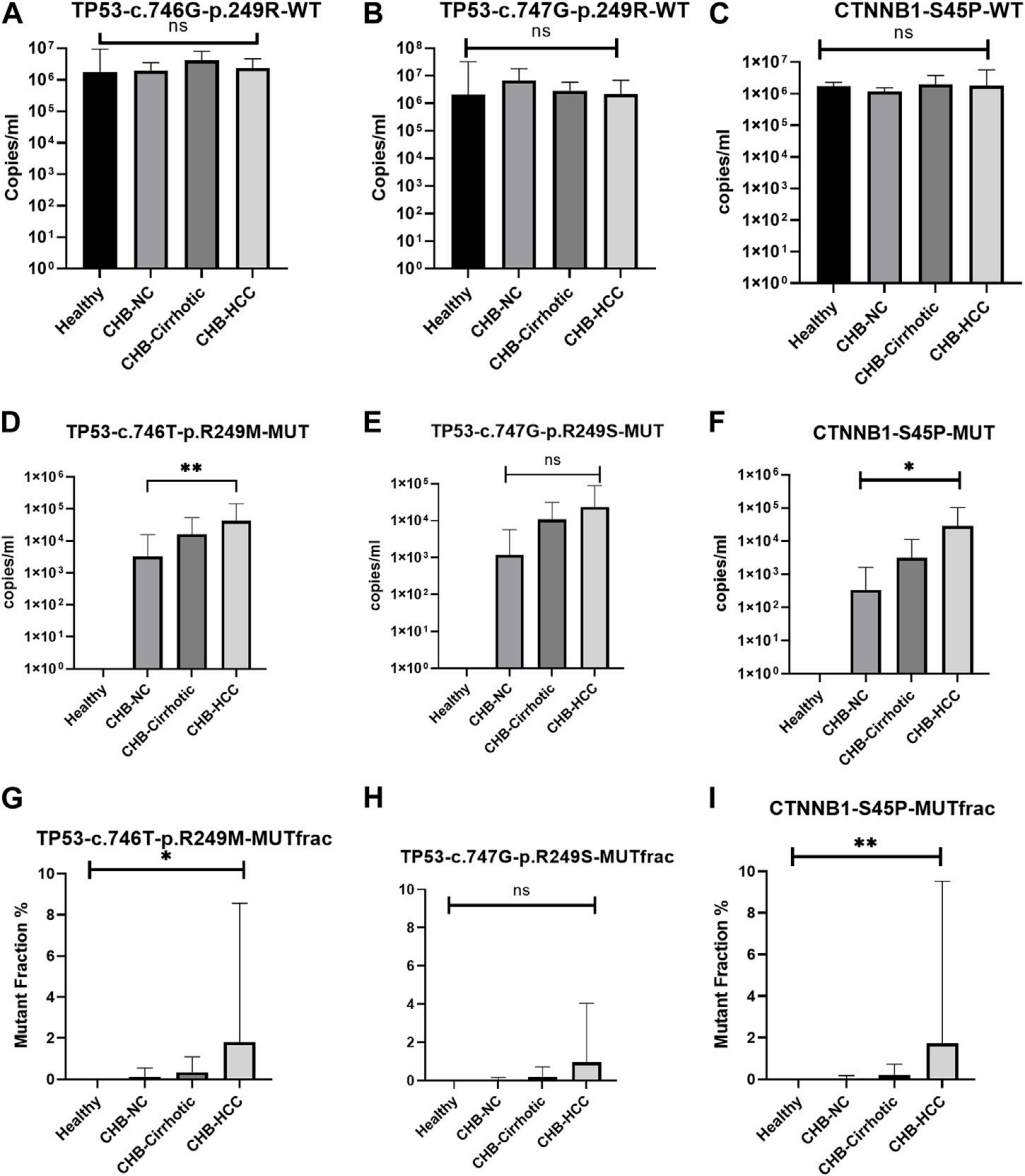
Wild type, mutant type copy number, and percentage mutant fraction for mutations TP53 R249M **(A–C)**, TP53 R249S **(D–F)**, and CTNNB1 S45P **(G–I)**. All the data was analysed using the one-way anova and Kruskal-Wallis tests. Multiple comparisons were done within groups, and the significance among the groups is depicted within the graphs.

There was no significant difference in wildtype copy number among healthy, CHB-cirrhotic, CHB-NC, and CHB-HCC patients (Table 4; Figures 3A–C). But a significant increasing trend of more TP53 and CTNNB1 mutant copy numbers was observed in CHB-HCC, followed by CHB-Cirrhotic, as compared to CHB-NC (Table 4; Figures 3D–F). The mutant fraction is the ratio of mutant-type and wild-type copies per mL of sample. The percentage mutant fraction has also shown an increasing trend, highest in CHB-HCC, followed by CHB-cirrhotic as compared to CHB-NC (Figures 3G–I). Mutant fraction was highest in HCC patients with a mean value of 1.81% for TP53 c.746G>T p. R249M, 0.95% for TP53 c.747GT p. R249S, and 1.73% for CTNNB1 c.133T>C S45P (Table 4).

### 3.5 Association of TP53 and CTNNB1 mutations with smoking, advanced stage, and mortality in HCC patients

In HBV-induced HCC patients, the odds ratios for the association of TP53 R249M, TP53 R249S, and CTNNB1 S45P with smoking, BCLC staging for advanced HCC, and mortality were calculated (Table 5). Smoking and the TP53 R249M mutation were found to be significantly associated (OR, 11.77; 95% CI, 3.219–36.20, *p* < 0.0001) with one another, but not with the TP53 R249S mutation.

**TABLE 5:** Association of TP53 R249M, TP53 R249S, and CTNNB1 S45P mutation with Smoking, BCLC staging and mortality.

	TP53 R249M		TP53 R249S		CTNNB1S45P	
Smoking	Yes/No	OR (95%CI); *p*-value	Yes/No	OR (95%CI); *p*-value	Yes/No	OR (95%CI); *p*-value
Smoker	12/4	11.77 (3.219–36.20) ***p* < 0.0001**	2/14	0.8730 (0.1737–4.282)	3/13	0.5897 (0.1645–2.298) *p* = 0.54
Non-Smoker	13/51		9/55	*p* > 0.99	18/46	

	TP53 R249M		TP53 R249S		CTNNB1S45P	
BCLC Staging	Yes/No	OR(95%CI); *p*-value	Yes/No	OR(95%CI); *p*-value	Yes/No	OR(95%CI); *p*-value
0+A+B	15/27	1.556 (0.6110–3.8) *p* = 0.4701	7/35	1.7	8/34	0.6588 (0.2156–1.823) *p* = 0.5928
C+D	10/28		4/34	0.4639–5.533, *p* = 0.5246	10/28	

	TP53 R249M and R249S		TP53 R249M and R249S and CTNNB1S45P			
BCLC Staging	Yes/No	OR(95%CI); *p*-value	Yes/No	OR(95%CI); *p*-value		
0+A+B	20/22	1.558, (0.6189–3.686), *p* = 0.3713	25/17	0.9591, (0.4111–2.293),P=>0.9999		
C+D	14/24		23/15			

	TP53 R249M		TP53 R249S		CTNNB1S45P	
Mortality	Yes/No	OR(95%CI); *p*-value	Yes/No	OR(95%CI); *p*-value	Yes/No	OR(95%CI); *p*-value
Dead	15/41	0.5122, (0.1949–0.330), *p* = 0.2005	9/47	2.106, (0.4622–10.30) *p* = 0.4904	15/41	1.098,0.3923–3.429 *p* > 0.9999
Alive	10/14		2/22		6/18	

Earlier studies had observed the association of the TP53 R249S mutation with aflatoxin exposure in CHB patients and the TP53 R249M association with smoking in lung cancer (Hollstein et al., 1991). The effect of these mutations on progression to advanced HCC (BCLC stages C and D) and mortality in patients was studied, but no significant association was observed for the TP53 and CTNNB1 genes (Table 5).

### 3.6 Effect of BCLC stage, smoking, and TP53, CTNNB1 mutations on survival of HCC patients

**FIGURE 4:**
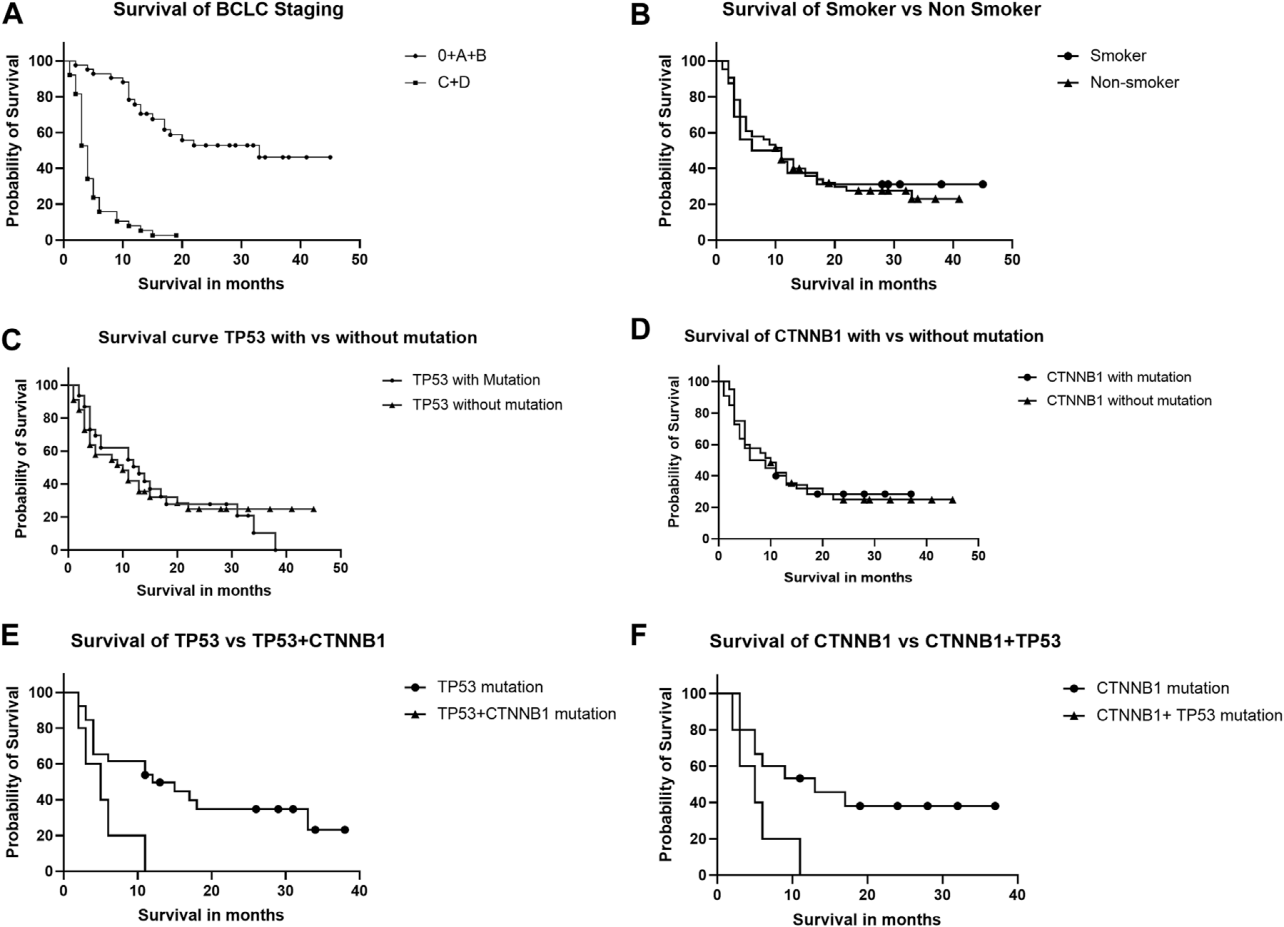
Kaplan-Meier curve analysis shows survival of HCC patients depending upon BCLC staging **(A)**, smoking **(B)**, TP53 and CTNNB1 mutations alone **(C,D)**, and in combination **(E,F)**.

The HCC patients were followed for survival for 48 months post-recruitment following the detection of TP53 and CTNNB1 mutations. Advanced HCC (BCLC stages C and D) was significantly associated with poorer survival (Figure 4A) than early HCC (BCLC stages 0, A, and B), as per the Kaplan-Meier survival curve [Hazard ratio (Mantel-Haenszel) HR = 14.19, 95% CI, 7.24–27.80, log-rank *p*-value < 0.0001).There was no significant difference in the probability of survival among those with and without smoking habits (Figure 4B), with or without TP53 (Figure 4C), or CTNNB1 (Figure 4D) mutations alone. The HCC patients with combined TP53 and CTNNB1 mutations had poorer survival than TP53 (Figure 4E) or CTNNB1 (Figure 4F) mutations alone with HR (Mantel-Haenszel), 6.253; 95% CI, 1.334–29.31; log-rank *p*-value, 0.02; and HR (Mantel-Haenszel), 5.660; 95% CI, 1.199–26.73; log-rank *p*-value, 0.02, respectively.

## 4 Discussion

Most hepatocellular carcinomas arise in the background of chronic liver disease (Sung et al., 2021). Viral infections due to HBV and HCV are major causes of chronic liver disease in the developing world, including India. The high mortality rate in HCC is mainly due to delayed diagnosis. Radiological imaging for screening of HCC is quite expensive for low-income populations (Dikshit et al., 2012; Valery et al., 2018). Alpha fetoprotein (AFP) is only a blood based detection biomarker, but not all HCC secretes AFP (Bialecki and Di Bisceglie, 2005). In the last decade, various pieces of evidence have demonstrated that liquid biopsy-based biomarkers, including cfDNA and ctDNA, have the potential for early disease diagnosis, disease progression prediction, prognosis, response to therapy, and personalised treatment (Diaz and Bardelli, 2014; Kumar et al., 2022). The role of ctDNA has been extensively studied in various malignancies, but its role in HCC diagnosis is still obscure. The establishment of ctDNA as a biomarker for HCC management requires extensive research and development (Bruix et al., 2014; Collins and Varmus, 2015). In this study, the driver mutations for cancer in the ctDNA of HCC and CHB patients were detected using highly sensitive ddPCR technology. The ctDNAs include a smaller fraction of cfDNA, which requires ultra-high sensitivity technologies to detect in the background of high cfDNA. Both NGS and ddPCR are modern technologies used for molecular profiling of ctDNA. The ddPCR is easy to use, economical, and able to detect very low mutant fractions (Hindson et al., 2011; Mouliere et al., 2011; Taylor et al., 2017). The driver mutations in the ctDNA of breast, colorectal, lung, and pancreatic cancers for the KRAS, PIK3CA, EGFR, and HER2 genes have been extensively studied by ddPCR assays. However, the driver mutations in these genes are not frequent in HCC (Higgins et al., 2012; Li et al., 2012; Taly et al., 2013; Oxnard et al., 2014; Shibata and Aburatani, 2014; Tantiwetrueangdet et al., 2018). The most frequently mutated genes in the HCC are TP53 and CTNNB1 genes (Huang et al., 2012; Cleary et al., 2013).

Hotspot mutations occur at evolutionarily conserved codons of TP53 in various types of cancer. The spectrum of mutations also differs among different cancers, including liver, lung, breast, brain, colon, esophagus, and blood cancer (Hollstein et al., 1991). Exposure to various mutagens, including ultraviolet light, aflatoxin B1, tobacco smoke (benzo [a]pyrene diol epoxide), and oncogenic viruses, also results in the formation of hotspot mutations (Baugh et al., 2018). Transversion-type (changes from purine bases G, C, to pyrimidine bases T, A) substitutions are most common for TP53 hotspot mutations in lung and liver cancer (Hollstein et al., 1991). Earlier studies have shown the most frequent transversion-type mutations at one nucleotide pair of TP53 codon 249 (c.747G>T, pR249S, AGG→AGT) in HCC patients of HBV aetiology from geographical areas of high dietary aflatoxin B1 exposure (Hollstein et al., 1991; Huang et al., 2016). But we did not observe the same mutation by sequencing in any of the HCC patients, but another transversion type mutation at TP53 codon 249, (c.746G>T, pR249M, AGG→ATG) was observed more frequently (25%) in HCC patients by sanger sequencing. Earlier, two studies from India reported an infrequent TP53 codon 249 (c.747G>T, pR249S) mutation in HBV-HCC, referring to low dietary aflatoxin exposure in India (Katiyar et al., 2000; Vivekanandan et al., 2011). Another study in Hong Kong of Chinese HBV-HCC patients from the low-exposure area for aflatoxin B1 has shown G-to- T transversions for codon 249 at locations c.746G>T, pR249M, and c.747G>T, pR249S (Ng et al., 1994). Another study by Wen et al. found that the TP53 mutation in HCC is an independent risk factor for the overall survival of the patients. However, the role of the TP53 mutation in HCC tumor recurrence is still unclear (Wen et al., 2016). Liu et al. (2012) observed in their meta-analysis that the TP53 mutation in HCC is associated with poor outcomes in patients. Such patients have a short, recurrence-free survival. This is the first study from India to report TP53 c.746G>T, pR249M mutations in HBV-induced HCC patients. This mutation, TP53 p.R249M, is quite common in lung cancer patients and is related to tobacco smoking exposure (Yokouchi et al., 2020). R249M is a missense somatic mutation most commonly observed in NSCLC tissue samples. In NSCLC, TP53 was found altered in approximately 50% of subjects, and about 0.3% of patients predominantly had the TP53 R249M mutation. Smokers and tobacco chewers have a high percentage of the TP53 p. R249M mutation (Halvorsen et al., 2016; Yokouchi et al., 2020). In Sanger sequencing of ctDNA, we observed that 4 out of 7 TP53 nsSNPs were found to be more pathogenic, with an evolutionary conservation score of 9 (H233R), 8 (Y234D), 9 (G245S), and 9 (R249M), respectively. All four residues are located within a stretch of the DNA binding domain, of which G245S and R249M are related to smoking exposure (Baugh et al., 2018). In the CTNNB1 gene, we observed six nsSNPs, and one nsSNP, S33T, was found to be more deleterious with a high (9) evolutionary conserved score.

In our study using a ddPCR assay, we observed that the TP53 c.746G>T, pR249M mutation is significantly higher in CHB-HCC patients (31.25%) than in CHB patients without HCC (14.28%). Whereas the TP53 c.747G>T and pR249S mutation frequencies were found to be similar and non-significant in both CHB patients with and without HCC. Overall, TP53 mutations were higher in HCC patients than in CHB patients without HCC. Similar observations were reported in another study by Marchio et al. (2018) in the African population. TP53 mutant fraction and frequency have shown a progressive increase from low in CHB-NC to high CHB cirrhotic and highest in HCC. The CTNNB1 p.S45P mutation observed in CHB and HCC patients was 11.4% and 26.50%, respectively. A high prevalence of p.S45P in the β-catenin gene in the ctDNA of HCC was also observed in other studies (Guichard et al., 2012; Ikeda et al., 2018; Howell et al., 2019).

The CTNNB1 mutant frequency was higher in HCC patients than CHB. The TP53 p.R249M mutation was not reported earlier in the ctDNA of HCC patients, but it was frequent in Indian HCC patients, as confirmed by both cycle sequencing and ddPCR assays. A significant association (OR, 11.77, 95% CI, 3.219–36.20, P 0.0001) of TP53 p.R249M with smoking was observed but no association for the TP53 p.R249S mutation in HBV-induced HCC (Table 5), and therefore cessation of smoking may be advised for CHB patients to avoid added risk exposure. In India, Global Adult Tobacco Survey-2 in the year 2016–1017 has observed 266.8 million tobacco consumers, of which smokeless tobacco (ST) consumers are 199.4 million and tobacco smokers are 99.5 million (Ministry of Health and Family Welfare, 2018). Smoking is considered independently a risk factor for HCC (Premkumar and Anand, 2021). The meta-analysis study have observed relative risk of 1.5 for in smoker as compared to non-smokers (Stepien et al., 2021). Other large meta-analysis study from Japan, found association of smoking with HCC (Tanaka et al., 2006). We observed that 57.5% of HCC patients had any single mutation, and 12.5% had any two gene mutations with a high mortality rate (Figure 4). In chronic liver disease patients, 31.4% had any mutation, while 11.4% had any two mutations. Similar results were observed by other research groups (Huang et al., 2016; Howell et al., 2019). We did not find any completely exclusive mutation that is contradictory to previous studies where the CTNNB1 mutation was exclusive to the TP53 mutation in the ctDNA of HCC patients (Huang et al., 2016).

The limitation of our study is the only inclusion of cirrhotic, noncirrhotic, and HCC patients of HBV etiology; therefore, findings may not be applicable to HCC of other etiologies. Tumor stage-specific mutation frequency was not studied in HCC patients. Paired tumour and peri-tumoral tissue samples were not studied for driver mutation validation as our goal was to evaluate molecular changes in ctDNA only. For smokers, we have not provided the smoking index (SI), which is calculated as per the formula SI = CPD (cigarettes per day) *years of tobacco use. We have collected only qualitative data (yes or no) (Feng et al., 2018). In India, tobacco products for smoking include both packed tobacco in cigarettes, cigars, and bidis and loose tobacco in hookahs or pipes. Conclusively, we have observed cancer driver mutations in the ctDNA using ddPCR technology with high precision and sensitivity. The ctDNA mutation was observed in advanced chronic liver disease patients (CHB-cirrhotic), so routine ctDNA screening of these high-risk patients may be helpful for identifying the risk of progression to HCC or early HCC detection and management. Hence, ctDNA has great potential to be a promising biomarker for early disease detection and to assess disease progression.

## Data Availability

The original contributions presented in the study are publicly available. This data can be found here: https://www.ncbi.nlm.nih.gov/genbank/. Accession Numbers: OR232241 - OR232272 (TP53); OR232273 - OR232304 (Beta-Catenin (CTNNB1)).
